# Concurrent antitumor and bone-protective effects of everolimus in osteotropic breast cancer

**DOI:** 10.1186/s13058-017-0885-7

**Published:** 2017-08-09

**Authors:** Andrew J. Browne, Marie L. Kubasch, Andy Göbel, Peyman Hadji, David Chen, Martina Rauner, Friedrich Stölzel, Lorenz C. Hofbauer, Tilman D. Rachner

**Affiliations:** 1 Division of Endocrinology, Diabetes and Bone Diseases, Department of Medicine III, Technical University Dresden, Fetscherstraße 74, D-01307 Dresden, Germany; 2 Center for Healthy Aging, Technical University Dresden, Dresden, Germany; 3 Philipps University of Marburg, Marburg, Germany; 4 Novartis Pharmaceutical Corp., East Hanover, NJ USA; 5 Division of Hematology, Department of Medicine I, Technical University Dresden, Dresden, Germany; 6 German Cancer Consortium (DKTK), partner site Dresden and German Cancer Research Center (DKFZ), Heidelberg, Germany

**Keywords:** mTOR inhibition, Breast cancer, Bone metastases, Hormone ablation, Antiresorptive

## Abstract

**Background:**

The mammalian target of rapamycin inhibitor everolimus is approved as an antitumor agent in advanced estrogen receptor-positive breast cancer. Surrogate bone marker data from clinical trials suggest effects on bone metabolism, but the mode of action of everolimus in bone biology remains unclear. In this study, we assessed potential bone-protective effects of everolimus in the context of osteotropic tumors.

**Methods:**

The effects of everolimus on cancer cell viability in vitro and on tumor growth in vivo were assessed. Everolimus-regulated osteoclastogenesis and osteoblastogenesis were also assessed in vitro before we assessed the bone-protective effect of everolimus in a model where bone loss was induced in ovariectomized (OVX) mice. Finally, the role of everolimus in the progression of osteolytic bone disease was assessed in an intracardiac model of breast cancer bone metastases.

**Results:**

At low concentrations (1 nM) in vitro, everolimus reduced the viability of human and murine cancer cell lines and impaired the osteoclastogenesis of osteoclast progenitors as assessed by quantitative real-time polymerase chain reaction and counting tartrate-resistant acid phosphatase-positive, multinucleated osteoclasts (*p* < 0.001). Everolimus had little or no deleterious effect on osteoblastogenesis in vitro, with concentrations of 1 and 10 nM increasing the messenger RNA expression of osteoblast marker genes (*p* ≤ 0.05) and leaving mineralization in differentiated human mesenchymal stem cells unchanged. Everolimus treatment (1 mg/kg body weight/day) prevented the bone loss observed in OVX mice and concurrently inhibited the metastatic growth of MDA-MB-231 cells by 70% (*p* < 0.002) while preserving bone mass in an intracardiac model of bone metastasis.

**Conclusions:**

These results underline the antitumor effects of everolimus and highlight its bone-protective efficacy, warranting further research on the potential implications on bone health in populations prone to osteoporosis and bone metastases, such as postmenopausal women with breast cancer.

**Electronic supplementary material:**

The online version of this article (doi:10.1186/s13058-017-0885-7) contains supplementary material, which is available to authorized users.

## Background

Maintaining bone health is a major clinical challenge in patients with breast cancer. Both the disease itself and most forms of treatment exert negative effects on bone metabolism [1, 2]. In particular, hormone-ablative treatment approaches in women with hormone receptor-positive cancers result in a rapid increase in bone resorption [3, 4]. In addition to the risk of osteoporosis, bone metastases are often seen as a late complication of apparently successfully treated patients with breast cancer [5], and novel antitumor agents are warranted that maintain bone health [6, 7].

The mammalian target of rapamycin (mTOR) signaling pathway is an important regulator of many cellular growth and disease processes [8]. Notably, activation of mTOR signaling is closely related to endocrine resistance in breast cancer [9–11]. The ability to overcome endocrine resistance was assessed in the pivotal phase III BOLERO-2 trial, where the mTOR inhibitor everolimus was assessed in postmenopausal women with estrogen receptor (ER)-positive breast cancer, whose disease progressed despite nonsteroidal aromatase inhibition using exemestane [12]. Exploratory analyses of bone turnover markers at 6 and 12 weeks revealed an expected increase in the exemestane cohort, but markers of bone turnover were significantly lower when combined with everolimus. In addition, the rate of metastatic bone disease was also lower in the combination group [13].

Of interest, a role for mTOR signaling has previously been attributed to different aspects of bone biology [14]. The activity of bone-resorbing osteoclasts depends on the mTOR pathway because the osteoclast differentiation factor receptor activator of nuclear factor κB ligand (RANKL) signals through the mTOR/p70 S6 kinase axis [15]. In osteoblasts, an increase of bone-protective osteoprotegerin (OPG) has been observed following mTOR inhibition by rapamycin [16], and in an ovariectomized (OVX) rat model, everolimus was shown to decrease osteoclast-mediated bone resorption and to inhibit the in vitro production of cathepsin K [17]. In this study, we sought to delineate the effects seen in the BOLERO-2 trial of everolimus-mediated mTOR inhibition on the individual cell types of the bone microenvironment in vitro, as well as in the context of the hormone-deprived environment often associated with osteolytic malignant bone disease in vivo.

## Methods

### Reagents and antibodies

Everolimus (RAD001) (catalogue number S1120) was purchased from Selleck Chemicals (Munich, Germany) and dissolved in dimethyl sulfoxide (DMSO). The following primary antibodies were purchased from Cell Signaling Technology, Inc. (Beverly, MA, USA): mTOR (catalogue number 2983), Phospho-mTOR (p-mTOR; catalogue number 2974), p70 S6 kinase (p70; catalogue number 9202), and phospho-p70 S6 kinase (p-p70; catalogue number 9205). An antibody for the housekeeping gene glyceraldehyde 3-phosphate dehydrogenase (GAPDH) (5G4) was purchased from HyTest Oy (Turku, Finland). HRP-conjugated mouse immunoglobulin G (IgG; catalogue number HAF007) and rabbit IgG (catalogue number HAF008) secondary antibodies were purchased from R&D Systems (Wiesbaden-Nordenstadt, Germany). Recombinant murine RANKL (catalogue number 462-TEC-010) and recombinant murine macrophage colony-stimulating factor (M-CSF; catalogue number 416-ML-010/CF) were purchased from R&D Systems (Minneapolis, MN, USA). Factors used to induce osteoblastic differentiation included dexamethasone (Sigma-Aldrich, Darmstadt, Germany), β-glycerol phosphate (Sigma-Aldrich), and ascorbate phosphate (Sigma-Aldrich). Recombinant human bone morphogenetic protein 2 (BMP-2; catalogue number 120-02) and BMP-4 (catalogue number 314-BP) used for murine osteoblast differentiation were purchased from PeproTech (Rocky Hill, NJ, USA). Calcein for labeling bone turnover was also purchased from Sigma-Aldrich.

### Cells and culture

Breast cancer cell lines MDA-MB-231 and MCF-7 and the murine melanoma cell line B16-F10 were purchased from the American Type Culture Collection (ATCC; Manassas, VA, USA). MDA-MD-231 cells transduced with the firefly luciferase gene (MDA-MB-231-LucA12) were a kind gift from Dr. Sanjay Tiwari (University of Kiel, Kiel, Germany). Breast cancer cells were cultured in Gibco DMEM/F-12 medium (Life Technologies GmbH, Darmstadt, Germany), and B16-F10 cells were cultured in 1× Gibco DMEM (Life Technologies GmbH).

RAW 264.7 cells (ATCC) and bone marrow-derived mononuclear cells derived from C57BL/6 mice were cultured in α-minimal essential medium (Biochrom, Berlin, Germany) supplemented with 2 mM glutamine (Biochrom). Cell cultures were maintained in a humidified atmosphere at 37 °C in 5% CO_2_/95% air atmosphere, and all culture medium conditions were supplemented with 10% fetal calf serum supreme (FCS; Biochrom) and Gibco 1% penicillin/streptomycin (P/S; Life Technologies GmbH). RAW 264.7 cells were seeded at a density of 1.25 × 10^4^ cells/cm^2^ and bone marrow-derived mononuclear cells at a density of 1 × 10^6^/cm^2^ when commencing osteoclast differentiation.

Mononuclear cells were isolated from donor bone marrow samples via density centrifugation before seeding in 1× DMEM supplemented with 10% FCS and 1% P/S. Nonadherent cells were removed 24 h later by washing with PBS. The resulting adherent human mesenchymal stem cells (hMSC) were cultured until confluency before seeding and again allowed to reach confluency before treatment. This study was approved by the local institutional review board ethics committee (EK245082010), and informed consent was obtained from healthy donors before bone marrow samples were collected at the Bone Marrow Transplantation Center of the University Hospital Carl Gustav Carus. Murine mesenchymal stem cells (mMSC) were isolated by flushing the bone marrow cells from the long bones of C57BL/6 mice into a culture of 1× DMEM. A medium change was performed 48 h later to remove nonadherent cells in a similar manner to the selection of hMSC. All adherent cell cultures were recovered using Gibco 0.25% trypsin-ethylenediaminetetraacetic acid (Life Technologies GmbH) before seeding and waiting until cells reached confluency before commencement of treatments.

### Osteoclast differentiation and tartrate-resistant acid phosphatase staining

Osteoclasts were differentiated from RAW 264.7 cells in the presence of 50 ng/ml murine RANKL and increasing concentrations of everolimus for 5 days with replacement of culture medium, murine RANKL, and everolimus taking place every 48 h. On day 5, cells were washed and fixed in acetone/citrate buffer. The Leukocyte Acid Phosphatase TRAP Kit (Sigma-Aldrich, Vienna, Austria) was used to stain cells for tartrate-resistant acid phosphatase (TRAP) according to the instructions of the manufacturer. Cells that were positive for TRAP staining and showing three or more nuclei were counted as osteoclasts, and representative photographs were captured for each treatment condition. Murine bone marrow-derived mononuclear cells sourced by bone marrow aspiration from the long bones of C57BL/6 mice were differentiated in 25 ng/ml murine M-CSF for 2 days before continuing with 25 ng/ml murine M-CSF and commencing 50 ng/ml murine RANKL and everolimus treatment for 5 days and performing TRAP staining as described for RAW 264.7 cells.

### In vitro bone resorption assay

Murine bone marrow-derived mononuclear cells were seeded onto bone slices (Immunodiagnostic Systems, Tyne & Wear, UK) with 25 ng/ml murine M-CSF and cultured for 2 days before medium was replaced with 25 ng/ml murine M-CSF and 50 ng/ml murine RANKL. Three days later, medium containing M-CSF and RANKL was replaced with the addition of everolimus. Everolimus-containing medium was changed 2 days later, and after a further 3 days of culture, supernatants were collected and analyzed for levels of collagen type I cross-linked C-telopeptide (CTx) using CrossLaps® for Culture (CTX-I) enzyme-linked immunosorbent assay (Immunodiagnostic Systems).

### Osteoblast differentiation and alizarin staining

hMSC or mMSC were seeded into a 24-well culture plate, and upon reaching confluency (on day 1), cells were treated with osteogenic differentiation medium (DMEM containing 10% FCS, and 1% P/S supplemented with 100 μM dexamethasone, 10 mM β-glycerol phosphate, and 100 μM ascorbate phosphate) with increasing concentrations of everolimus. On day 21, cells were washed twice with PBS and fixed with 10% paraformaldehyde (PFA) for 15 minutes at room temperature. Following fixation, cells were washed twice with distilled water and incubated in a 40 mM alizarin red S solution, pH 4.2 (Sigma-Aldrich, Munich, Germany) for 20 minutes at room temperature. Stained cells were subsequently washed with distilled water until the excess dye was completely removed. Plates were dried at room temperature overnight before imaging. To quantify mineralization, the alizarin red S bound to the mineralized calcium was eluted in 0.1 M HCl/0.5% sodium dodecyl sulfate (SDS) solution for 30 minutes at room temperature. The resulting eluent was measured with a spectrophotometer at 540 nm. Each treatment was performed in triplicate for three different donors. In the murine osteoblast experiments, 100 ng/ml of BMP-2 and BMP-4 was added to the culture conditions.

### Cell viability assay

Cancer cell lines and bone cells (differentiated RAW 264.7 cells and hMSC) were seeded onto 96-well and 24-well plates, respectively. The CellTiter-Blue® assay (Promega, Mannheim, Germany) was used according to the manufacturer’s instructions to evaluate cell viability at different time points following everolimus treatments at different concentrations (0, 1, 10, and 100 nM). Cancer cells were seeded at a density of 3000 cells per 96-well plate. hMSC were seeded at a density of 60,000 cells per 24-well plate and allowed to reach confluency before differentiation for 8 days and assessment of the effects of everolimus concentrations only after a treatment duration of 48 h. RAW 264.7 cells were seeded at a density of 25,000 cells per 24-well plate and differentiated with 20 ng/ml murine RANKL before being treated with everolimus for 48 h.

### RNA isolation, reverse transcription, and real-time polymerase chain reaction

Polymerase chain reactions (PCRs) were performed as previously described [18]. Briefly, the High Pure RNA Extraction Kit (Roche Applied Science, Mannheim, Germany) was used to isolate RNA following the manufacturer’s protocol. Purified RNA (500 ng) was reverse-transcribed using SuperScript II (Life Technologies GmbH) and underwent SYBR Green-based real-time polymerase chain reaction (RT-PCR) using a standard protocol (Applied Biosystems, Foster City, CA, USA). Primer sequences are listed in Table 1. The PCR cycling program ran at 50 °C for 2 minutes and at 95 °C for 10 minutes, followed by 40 cycles at 95 °C for 15 seconds and 60 °C for 1 minute. The melting curve was assessed at 95 °C for 15 seconds, 60 °C for 1 minute, and 95 °C for 30 seconds. The comparative cycle threshold method was used to calculate the results, which are presented as the *x*-fold increase relative to the housekeeping gene (human β-actin or murine β-actin) or as a percentage of the control.

**Table 1:** Primers used for real-time quantitative reverse transcription-polymerase chain reaction

Targeted gene	Primer sequences (5′-3′)
*ACTB*	CCAACCGCGAGAAGATGA
	CCAGAGGCGTACAGGGATAG
*ALP*	CAACCCTGGGGAGGAGAC
	GCATTGGTGTTGTACGTCTTG
*OPG*	GAACCCCAGAGCGAAATACAG
	TAGCAGGAGACCAAAGACACTG
*RUNX2*	CAGATGGGACTGTGGTTACTG
	TGGGGAGGATTTGTGAAGAC
*OCN*	TGAGAGCCCTCACACTCCTC
	ACCTTTGCTGGACTCTGCAC
*Actb*	GATCTGGCACCACACCTTCT
	GGGGTGTTGAAGGTCTCAAA
*Alp*	CTACTTGTGTGGCGTGAAGG
	CTGGTGGCATCTCGTTATCC
*Opg*	CCTTGCCCTGACCACTCTTA
	ACACTGGGCTGCAATACACA
*Runx2*	CCCAGCCACCTTTACCTACA
	TATGGAGTGCTGCTGGTCTG
*Ocn*	GCGCTCTGTCTCTCTGACCT
	ACCTTATTGCCCTCCTGCTT
*Rankl*	CACTGAGGAGACCACCCAAG
	GAGATGAAGAGGAGCAGAACG
*Oscar*	TCTGCCCCCTATGTGCTATC
	CTCCTGCTGTGCCAATCAC
*Trap*	ACTTGCGACCATTGTTAGCC
	AGAGGGATCCATGAAGTTGC
*Ctsk*	AAGTGGTTCAGAAGATGACGGGAC
	TCTTCAGAGTCAATGCCTCCGTTC

*Abbreviations: ACTB/Actb* β-Actin, *ALP/Alp* Alkaline phosphatase, *Ctsk* Cathepsin K, *OCN/Ocn* Osteocalcin, *OPG/Opg* Osteoprotegerin, *RANKL/Rankl* Receptor activator of nuclear factor-κB ligand, *RUNX2/Runx2* Runt-related transcription factor 2, *TRAP/Trap* Tartrate-resistant acid phosphatase

### Immunoblotting

The analysis of protein expression by Western blotting was performed as described previously [19]. In short, following treatment with everolimus, cancer cells were lysed and protein levels quantified. Protein samples of 20 μg were loaded onto a 6% SDS-PAGE gel and separated by electrophoresis. The separated proteins were then transferred onto a 0.2-μm nitrocellulose membrane. Blocking was performed in 5% nonfat dry milk in Tris-buffered saline with 1% Tween 20 for 1 h. Membranes were then washed in Tris-buffered saline with 1% Tween 20 and incubated overnight in 5% bovine serum albumin in Tris-buffered saline with 1% Tween 20 containing the primary antibody (mTOR, phosphorylated mTOR, p70 S6 kinase, phosphorylated p70 S6 kinase, or GAPDH). Membranes were washed before incubation for 1.5 h with the HRP-conjugated secondary antibody in 1% nonfat dry milk in Tris-buffered saline with 1% Tween 20. After another washing step, the membranes were developed and the protein visualized using SuperSignal substrate (Pierce Biotechnology, Bonn, Germany) enhanced chemiluminescence. Phosphorylated protein signals were quantified and normalized to GAPDH signals using ImageJ version 1.44 software (imagej.nih.gov/ij/).

### Animal experiments

Female immunocompromised NMRI nude and immunocompetent C57BL/6 mice were housed under institutional guidelines. The institutional animal care committees of the Technical University Dresden and the Landesdirektion Dresden approved all animal procedures (IRB TVV 61/2015). In subcutaneous tumor models, NMRI nude and C57BL/6 mice were inoculated subcutaneously at 6 weeks of age with 1 × 10^6^ MDA-MB-231 and 1 × 10^4^ B16-F10 cells, respectively, in 50 μl of a 1:1 Matrigel matrix dilution with PBS on day 1. The site of subcutaneous inoculation was dorsal, between the positions of the last rib and the hind limb, with two injection sites per mouse, left and right. Intraperitoneal injections (100 μl) of 1 mg/kg/day everolimus or control DMSO commenced on day 2 for 4 weeks in the case of the MDA-MB-231 model and for 2 weeks in the case of the B16-F10 model before mice were killed to assess tumor burden. Ten mice were allocated to each treatment group. To establish a model of bone loss similar to that of the human condition, an estrogen-deprived environment was induced in the C57BL/6 strain by performing OVX in 9-week-old mice. Four weeks postsurgery, OVX or sham (SHAM)-operated groups were treated intraperitoneally with 1 mg/kg/day everolimus or control for 4 weeks. Ten mice were allocated to each group initially. However, two mice did not survive the OVX procedure, resulting in both SHAM groups having only nine animals each at the time mice were killed. Calcein (15 mg/kg) labeling was performed 5 and 2 days before mice were killed. Killing was done after 8 weeks. In the bone metastasis model, 1 × 10^5^ MDA-MB-231-LucA12 cells were injected into the left ventricle of the heart of 6-week-old NMRI nude mice under ultrasound guidance. Intraperitoneal injections of 100 μl, 1 mg/kg/day everolimus or control DMSO, commenced on the day of tumor cell inoculation. Ten mice per group were initially included. One mouse from each group died following anesthetic administration at the first imaging session and were therefore excluded from the experiment. No adverse effects were observed for treatment with everolimus in any of the experiments performed.

### Bone assessment

Micro-computed tomography (μCT, vivaCT 75; SCANCO Medical, Brüttisellen, Switzerland) was performed on the excised femurs using X-ray energy of 70 keV, a resolution of 10.5 μm, and an integration time of 200 milliseconds. Calibration of the scanner took place weekly using hydroxyapatite (HA) phantoms. For the 3D visualization of bony tissue, we used the SCANCO evaluation software (SCANCO Medical). The threshold for bone absorption values was set to 285 mg HA/cm^3^, and 100 slices were measured commencing from 10 slices above the growth plate of the femur.

### Bone histomorphometric analysis

The femur was fixed in 4% PFA/PBS for 24 h, dehydrated in an ascending ethanol series, and embedded in paraffin. The tibia was separately embedded in methyl methacrylate (Technovit 9100 NEW; Heraeus, Wehrheim, Germany). Sections (2 μm) were used to stain for TRAP in the femur, allowing for the assessment of the number of osteoclasts per unit of bone surface. Sections (7 μm) were cut from the tibia for the assessment of calcein labels to determine the bone formation rate per unit of bone surface (BFR/BS). Analysis and quantification of the bone histomorphometric parameters were performed using OsteoMeasure software (OsteoMetrics, Decatur, GA, USA). Relevant units for histomorphometric measurements were consistent with those advised by the nomenclature committee of the American Society for Bone and Mineral Research.

### Bioluminescence imaging and quantification

Bioluminescence imaging was used to quantify tumor growth by correlating the tumor burden to the luminescence signal measured with a Xenogen IVIS 200 in vivo imaging system (PerkinElmer, Rodgau, Germany). Successful intracardiac injection was determined immediately after intracardiac inoculation. Imaging for the assessment of tumor growth commenced 2 weeks postinoculation, and continuous assessment was performed once weekly until mice were killed at the end of week 5. Living Image software (PerkinElmer) was used to obtain and quantify the bioluminescence data. Mice were anesthetized, and 5 minutes prior to imaging, each mouse was given an intraperitoneal injection with a dose of 10 mg/kg D-luciferin (PerkinElmer) in PBS. Mice were imaged individually for an exposure period of 2 minutes. The resulting bioluminescent images were analyzed by measuring individual, manually contoured signals with final measurement units in photons per second per centimeter squared per steradian.

### Statistical analyses

Each in vitro experimental setup was repeated a minimum of three times, and using Prism 6 software (GraphPad Software, Inc., La Jolla, CA, USA), one-way analysis of variance (ANOVA) with the Bonferroni posttest or Student’s *t* test was performed to evaluate the equality of the mean. To analyze the effects of OVX and everolimus treatment, two-way ANOVA with Tukey’s posttest was performed. The results are presented as SD of the mean, and a *p* value <0.05 was considered statistically significant.

## Results

### Effects of everolimus on cancer growth in vitro and in vivo

**Fig. 1:**
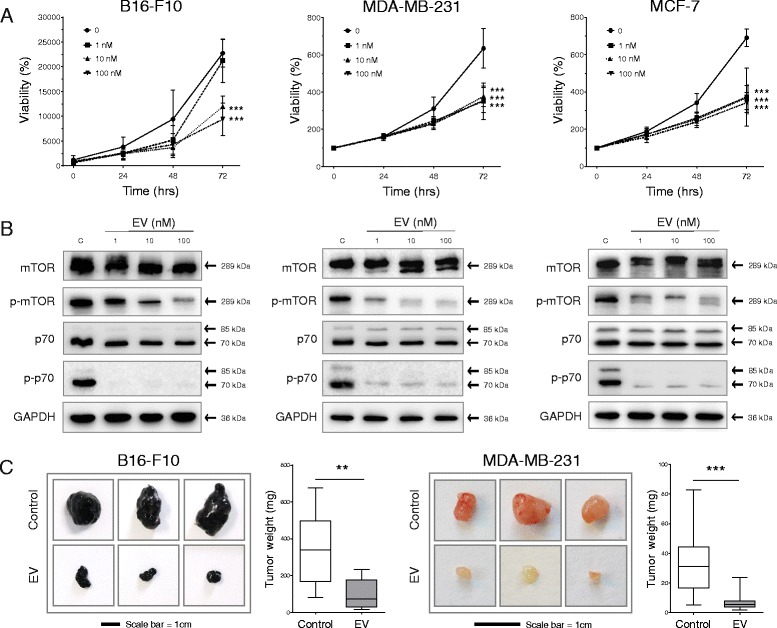
Everolimus (EV) inhibits cancer cell growth in vitro and in vivo. **a** The murine melanoma cell line B16-F10 and the human breast cancer cell lines MCF-7 and MDA-MB-231 were treated with EV in a dose-dependent (0, 1, 10, and 100 nM) and time-dependent (0, 24, 48, and 72 h) manner. Cell viability was assessed with the CellTiter-Blue® assay. **b** Western blots used to assess the ability of EV concentrations to inhibit the phosphorylation of the mammalian target of rapamycin (mTOR) protein and p70 S6 kinase after 24 h of treatment in the cell lines investigated. Glyceraldehyde 3-phosphate dehydrogenase (*GAPDH*) is shown as the housekeeping control. **c** Female immunocompetent (C57BL/6) and immunocompromised (NMRI nude) mice were inoculated subcutaneously with B16-F10 and MDA-MB-231 cells, respectively. Tumor growth was assessed after daily treatment with 1 mg/kg of EV for 2 and 4 weeks in each respective model. In vitro and in vivo data are shown as mean ± SD of at least three independent experiments or ten mice per group, respectively. Cell viability assays were analyzed for each time point using two-way analysis of variance and in vivo data by Student’s *t* test. Significance between EV treatments and the control condition was apparent only at 72 h and is indicated by asterisks on the graphs. In the B16-F10 graph, EV treatment was significant at inhibiting viability only at 72 h at concentrations of 10 and 100 nM. In the MDA-MB-231 and MCF-7 graphs, all concentrations of EV were significant to the same degree. ** *p* < 0.01, *** *p* < 0.001. Equal volumes of dimethyl sulfoxide used to prepare and administer EV treatments were used in both in vitro and in vivo control conditions

In the B16-F10, MDA-MB-231, and MCF-7 cell lines, everolimus exerted a potent negative effect on the growth of all cell lines tested as assessed by the CellTiter-Blue cell viability assay (Fig. 1a). In the murine B16-F10 melanoma cell line, concentrations of 10 and 100 nM were effective at significantly impairing cell viability when assessed at 72 h (*p* < 0.05 and 0.01, respec tively). In the human breast cancer cell line MDA-MB-231, everolimus at a concentration as low as 1 nM was sufficient to induce significant suppression in viability, most notably after 72 h of treatment, when a 45% reduction in viability was observed compared with control treated cells (*p* < 0.05). Antitumor effects of everolimus were also apparent and equally effective for all three concentrations used in the ER-positive MCF-7 cell line. Effective inhibition of the mTOR pathway was confirmed by Western blot assessment of mTOR phosphorylation and the downstream target of mTOR, p70 S6 kinase (Fig. 1b). Increasing everolimus concentrations inhibited the phosphorylation of mTOR in a dose-dependent manner, with 100 nM inducing a significant suppression in all three cell lines investigated (*p* < 0.01) (Additional file 1: Figure S1). Interestingly, all concentrations were sufficient to significantly suppress the phosphorylation of p70 S6 kinase by ≥50% (*p* < 0.01) (Additional file 1: Figure S1). In murine models of subcutaneous tumor growth, everolimus at a dose of 1 mg/kg/day was sufficient to significantly inhibit the growth of B16-F10 and MDA-MB-231 cells over a period of 2 and 4 weeks for each respective tumor model. Tumor weight was reduced by 71% (345 ± 66 mg to 103 ± 25 mg, *p* < 0.01) and 81% (34 ± 5 to 7 ± 1 mg, *p* < 0.001) in the B16-F10 and MDA-MB-231 models, respectively (Fig. 1c).

### Effects of everolimus on osteoclast differentiation

**Fig. 2:**
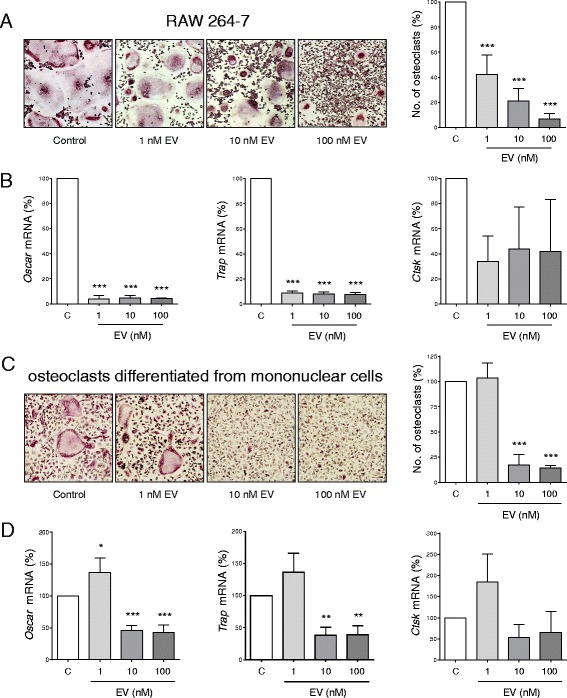
Effects of everolimus (EV) on osteoclastogenesis of osteoclast progenitor RAW 264.7 cells and murine bone marrow-derived mononuclear cells in vitro. **a** RAW 264.7 osteoclast precursors were treated with receptor activator of nuclear factor κB ligand (RANKL) in the presence of increasing EV concentrations (0, 1, 10, and 100 nM) for 5 days. Differentiation was assessed by tartrate-resistant acid phosphatase (TRAP) staining and counting. **b** Osteoclast marker genes *Cstk*, *Oscar*, and *Trap* were assessed by quantitative real-time polymerase chain reaction (qRT-PCR) following differentiation with RANKL and EV for 5 days. **c** Murine bone marrow-derived mononuclear cells were isolated from the bone marrow of C57BL/6 mice and treated with macrophage colony-stimulating factor (M-CSF) for 2 days prior to further M-CSF plus the addition of RANKL in the presence of increasing EV concentrations (1–100 nM) for 5 days. Differentiation was assessed by TRAP staining and counting. **d** Osteoclast marker genes *Cstk*, *Oscar*, and *Trap* were also assessed for osteoclasts differentiated from bone marrow-derived mononuclear cells by qRT-PCR following differentiation with RANKL and EV for 5 days. Data are shown as mean ± SD of at least three independent experiments. Data were analyzed using one-way analysis of variance and the Bonferroni posttest, and significance between the control and EV concentrations is denoted (* *p* < 0.05, ** *p* < 0.01, *** *p* < 0.001). Equal volumes of dimethyl sulfoxide used to prepare and administer EV concentrations were used in all control conditions. *mRNA* Messenger RNA

RAW 264.7 osteoclastic precursor cells predifferentiated with RANKL for 5 days and exposed to everolimus for 48 h showed a significant reduction of cell viability with incremental decreases of 13%, 21%, and 28% for the increasing concentrations of 1, 10, and 100 nM everolimus, respectively (Additional file 2: Figure S2a). In addition, everolimus exerted potent negative effects on osteoclast formation. The number of TRAP-positive cells developing in the presence of RANKL was reduced by 58% at an everolimus concentration of 1 nM (*p* < 0.001) (Fig. 2a). In accordance with this, markers of osteoclast differentiation, including *Trap* and *Oscar*, were significantly reduced by all concentrations of everolimus (*p* < 0.001) (Fig. 2b). There was a trend that the expression of *Cstk* was also reduced at all everolimus concentrations; however, this observation was not significant. Comparable effects of everolimus were observed in osteoclasts differentiated from murine bone marrow-derived mononuclear cells, where concentrations of 10 nM were sufficient to completely block osteoclastogenesis and expression of osteoclast marker genes (Fig. 2c and d). Of note, primary murine cells were more resistant to the lowest concentration of 1 nM everolimus, where no inhibitory effects were observed on osteoclast differentiation. Osteoclasts derived from primary murine cells were also used to assess the effect of everolimus on functional bone resorption by mature osteoclasts in vitro. Here, mononuclear cells were differentiated into osteoclasts on bone slices, and after a treatment period of 5 days with everolimus, it could be clearly observed that concentrations of 10 and 100 nM significantly decreased the levels of the bone resorption marker CTx present in the culture supernatants by 34.2% and 33.4% (*p* < 0.01), respectively (Additional file 3: Figure S3).

### Effects of everolimus on osteoblast differentiation

**Fig. 3:**
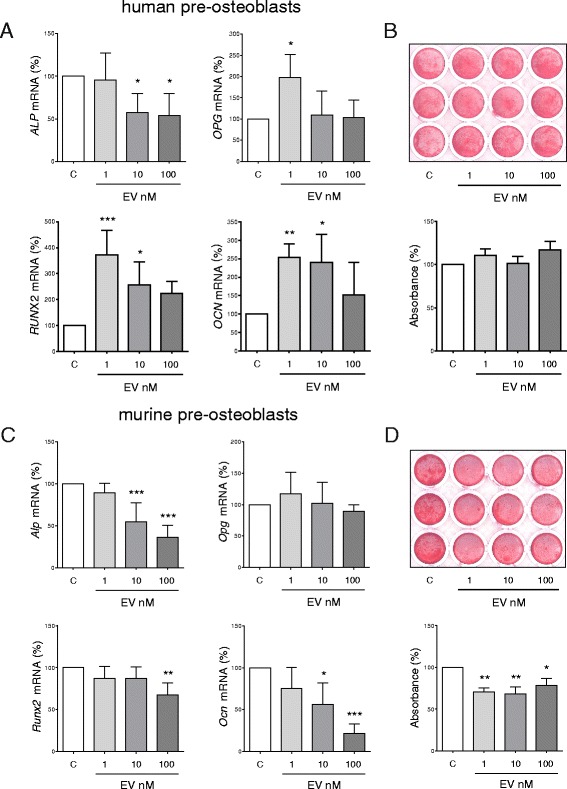
Effects of everolimus (EV) on human and murine osteoblastogenesis in vitro. Human mesenchymal stem cells were differentiated along the osteoblast lineage in the presence of increasing EV concentrations (0, 1, 10, and 100 nM). **a** Osteoblast marker genes *ALP*, *OPG*, *RUNX2*, and *OCN* were assessed by quantitative real-time polymerase chain reaction (RT-PCR) at 7 days of differentiation. **b** The mineralizing ability of these cells was quantified using alizarin red S staining on day 21. Murine mesenchymal stem cells were isolated from the bone marrow of C57BL/6 mice and differentiated along the osteoblast lineage in the presence of increasing EV concentrations (1–100 nM). **c** Osteoblast marker genes *Alp*, *Opg*, *Runx2*, and *Ocn* were assessed by qRT-PCR at 7 days of differentiation. **d** The mineralizing ability of these cells was quantified using alizarin red S staining on day 21. Data are shown as mean ± SD of at least three independent experiments. Data were analyzed using one-way analysis of variance and the Bonferroni posttest, and significance between the control and EV concentrations is denoted (* *p* < 0.05, ** *p* < 0.01, *** *p* < 0.001). Equal volumes of DMSO used to prepare and administer EV concentrations were used in all control conditions. *mRNA* Messenger RNA

In human preosteoblasts derived from hMSC, markers of osteoblastogenesis *ALP*, *OPG*, *RUNX2*, and *OCN* were quantified by qRT-PCR on day 7 following the addition of osteoblastic differentiation medium and increasing everolimus concentrations (Fig. 3a). Here, the messenger RNA expression of *ALP*, a reliable marker of osteoblast activity, was negatively affected only at higher concentrations of everolimus (10 and 100 nM). In fact, the expression of *OPG*, *RUNX2*, and *OCN* significantly increased at 1 nM and remained elevated at 10 nM for *RUNX2* and *OCN*. No inhibitory effect of everolimus on mineralization as assessed by alizarin red staining was observed after 21 days of exposure to everolimus at concentrations of up to 100 nM (Fig. 3b). When assessing the same parameters in murine preosteoblasts derived from mMSC, we observed that the expression of osteoblast marker genes following differentiation for 7 days in the presence of everolimus showed increasing reductions in *Alp* and *Ocn* from a concentration of 10 nM and only at 100 nM for *Opg* and *Runx2* (Fig. 3c). Whereas 1 nM of everolimus maintained the osteoblast expression profile of all the genes assessed, we could not observe any pro-osteoblastic effects as seen in the osteoblastic gene signature of differentiating hMSC. When the mineralizing ability of everolimus-treated murine osteoblasts was assessed at 21 days, a significant decrease of 29% between control treated cells and cells treated with 1 nM of everolimus was observed (*p* < 0.01). However, increasing concentrations of everolimus did not result in further impairment of mineralization. When investigating whether everolimus has implications on osteoblastic metabolism, we observed that concentrations increasing from 1 nM to 100 nM did not show any effects on the viability of human preosteoblasts (Additional file 2: Figure S2b)

### Effect of everolimus in an OVX murine model of bone loss

**Fig. 4:**
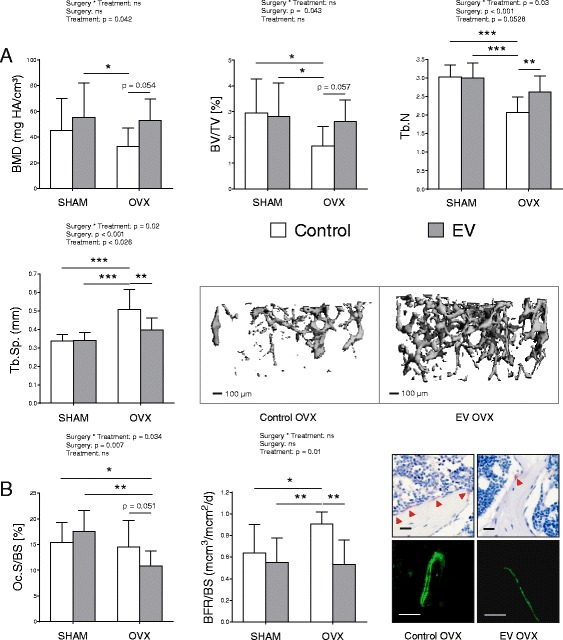
Everolimus (EV) protects ovariectomized mice from bone loss. Female C57BL/6 mice were divided into sham (SHAM) and ovariectomized (OVX) groups and subdivided into control or 1 mg/kg/day EV treatment groups (eight to ten mice per group). Four weeks post-OVX, treatment with EV commenced for 4 weeks. Bone parameters of the femur were assessed by micro-computed tomography (μCT) (**a**), and bone parameters of the tibia were assessed by bone histomorphometry (**b**). Parameters assessed included bone mineral density (BMD), bone volume over total volume (BV/TV), trabecular number (Tb.N), and trabecular separation (Tb.Sp). The number of osteoclasts per unit of bone surface (Oc.N/BS) was assessed by tartrate-resistant acid phosphatase (TRAP) staining (femur), and assessment of the double calcein labels (tibia) was used to determine the bone formation rate per unit of bone surface (BFR/BS). Representative μCT images are shown of the trabecular bone of the femur for the control OVX and EV OVX groups. Representative TRAP staining (with *red arrowheads* indicating osteoclasts) (original magnification × 40, scale bar 20 μm) and double calcein labels for these two groups are also provided (original magnification × 20, scale bar 100 μm). Data represent mean ± SD. Statistical analysis was performed by two-way analysis of variance for the effect of surgery, treatment, and the interaction of the two (surgery × treatment). Statistical significance of multiple comparisons are denoted (* *p* < 0.05, ** *p* < 0.01, *** *p* < 0.001). Equal volumes of dimethyl sulfoxide used to prepare and administer EV concentrations were used in all control conditions. *HA* Hydroxyapatite

Because the majority of patients with breast cancer are postmenopausal when they present for diagnosis, or undergo hormone depletion therapies over the course of treatment, we wanted to recapitulate the hormone-deprived microenvironment in an animal model. To this end, 9-week-old wild-type C57BL/6 mice underwent OVX to induce an environment of high bone turnover and bone loss. Treatment with everolimus commenced 4 weeks post-OVX, and mice were treated with 1 mg/kg/day. Assessment of bone was performed after 4 weeks of treatment. As expected, there was a decrease in bone mineral density (BMD) in the OVX group compared with the SHAM-operated group by 27.49% (45.00 ± 24.85 vs. 32.63 ± 14.58) at the femur. Treatment with everolimus had a significant effect on BMD, restoring OVX-induced bone loss (Fig. 4a). BMD of the everolimus-treated OVX group was 38.44% (53.00 ± 16.57 vs. 32.63 ± 14.58) higher than that of the OVX control group. The average bone volume over total volume (BV/TV) of everolimus-treated OVX mice was also 37% higher than in the control OVX mice (2.62 ± 0.85 vs. 1.67 ± 0.75). These results were echoed by an increase in trabecular number (2.62 ± 0.43 vs. 2.07 ± 0.41, *p* < 0.01) and a decrease in trabecular separation (0.40 ± 0.07 vs. 0.51 ± 0.11, *p* < 0.01) in everolimus-treated OVX mice versus control OVX mice (Fig 4a). Analysis of bone histomorphometry demonstrated a 25% reduction in the number of osteoclasts in contact with the bone surface (14.54 ± 5.08 to 10.87 ± 2.89) in the everolimus-treated OVX mice when compared with control OVX mice (Fig. 4b), also depicted as whole sections (Additional file 4: Figure S4). Correspondingly, the BFR/BS increased in control OVX mice by 30% (from 0.64 ± 0.26 to 0.91 ± 0.11) when compared with the rate of control SHAM animals, and everolimus was able to reverse this increase by 41.5% (0.91 ± 0.11 to 0.53 ± 0.23, *p* < 0.01) (Fig. 4b). This demonstrates that everolimus prevents the high bone turnover and bone loss that is induced by OVX.

### Effects of everolimus on growth of bone metastases

**Fig. 5:**
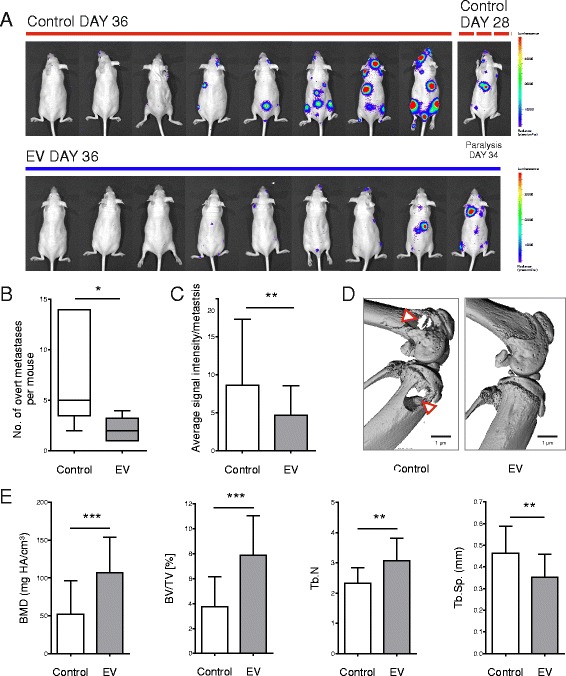
Everolimus (EV) inhibits growth of breast cancer bone metastases in vivo. **a** Female NMRI nude mice received intracardial injections with MDA-MB-231 cells expressing the firefly luciferase gene. Mice received daily treatments of control (nine mice) or 1 mg/kg EV (nine mice), and developing metastases were monitored weekly using the Xenogen IVIS 200 in vivo imaging system until mice were killed on day 36. Of note, one mouse in the control group died early as a result of paralysis on day 34. For this mouse, the measurement on day 28 was included. No animals in the EV treatment group developed paralysis. Representative dorsal-facing images with the observed bioluminescent signal at sites of tumor burden are shown, with animals arranged from *left* to *right* according to increasing bioluminescent signals. **b** The number of lesions per animal with signals ≥1 × 10^7^ photons/s/cm^2^/sr were counted and compared between the groups, and the results are presented in a box plot. **c** The average luciferase signal intensity (per second per centimeter squared per steradian) from regions of interest was calculated per metastatic signal focus (EV *n* = 57 detectable lesions, control *n* = 90 detectable lesions). **d** The sites of bioluminescent signal in the knee joint were confirmed by 3D micro-computed tomography (µCT) and corresponded with osteolysis (as indicated by *red and white arrowheads*). **e** Bone parameters of the femur where assessed by μCT: bone mineral density (BMD), bone volume over total volume (BV/TV), trabecular number (Tb.N), and trabecular separation (Tb.Sp). Data are shown as mean ± SD and were analyzed using Student’s *t* test (* *p* < 0.05, ** *p* < 0.01, *** *p* < 0.001). Equal volumes of dimethyl sulfoxide used to prepare and administer EV concentrations were used in all control conditions. *HA* Hydroxyapatite

Having established the bone-protective effects of everolimus at a concentration capable of exerting antitumor potential, the ability of everolimus to inhibit the development of osteolytic breast cancer bone metastases was assessed. Firefly luciferin-labeled MDA-MB-231 breast cancer cells (MDA-MB-231-LucA12) were intracardially injected into 6-week-old NMRI nude mice. Images of all injected mice with the observed bioluminescent signal at sites of tumor burden are shown prior to their being killed at day 36 (Fig. 5a). The number of overt lesions per mouse in animals with bioluminescent signals >1 × 10^7^ photons/second/cm^2^/sr were counted and compared between the groups. Everolimus-treated animals had a significantly reduced number of overt lesions compared with control animals (−70.4%, 7.33 ± 5.32 to 2.17 ± 1.17, *p* < 0.05) (Fig. 5b). Each metastatic signal was also individually quantified, and this was reduced by 45.4% (8.62 ± 8.69 to 4.71 ± 3.86, *p* < 0.01) in the everolimus group compared with the control group (Fig. 5c). Osteolytic lesions corresponded with bioluminescent signals and could be visualized by reconstructing μCT scans of analyzed bones. Representative images of affected and unaffected femurs and tibiae are shown (Fig. 5d). Trabecular bone quality in the femurs of these animals was assessed by μCT analysis (Fig. 5e). Animals in the everolimus-treated group had an increased BMD and BV/TV of >50% (*p* < 0.001) compared with the placebo-treated group. Trabecular parameters reflected this observation, with the control group having 24.25% (2.33 ± 0.52 to 3.07 ± 0.75, *p* < 0.01) less trabeculae and 31.52% (0.46 ± 0.12 to 0.35 ± 0.11, *p* < 0.01) more separation between the trabeculae than the everolimus group. Interestingly, the total number of osteoclasts in the femurs of everolimus-treated mice were decreased by 42.88% (98.30 ± 39.21 to 58.11 ± 19.68, *p* < 0.05) compared with control-treated mice (Additional file 5: Figure S5). This experiment confirmed combined antiosteoclastic and antitumor effects of everolimus in the metastatic bone microenvironment.

## Discussion

Most hormone-ablative breast cancer therapies decrease patients’ bone density and integrity by increasing osteoclastogenesis and bone resorption [2, 20]. This is especially the case for aromatase inhibitors, which are now standard of care for postmenopausal patients with ER-expressing breast cancer. In the BOLERO-2 trial, mTOR inhibition using everolimus exerted a clinical benefit to patients with ER-positive breast cancer progressing despite hormone therapy [12], and exploratory assessment of markers of bone turnover from this trial revealed a lower increase in the group receiving everolimus in combination with aromatase inhibitors than aromatase inhibitor treatment alone. However, bone turnover reflects only one aspect of bone biology and does not necessarily correlate with bone density. Researchers in a number of preclinical studies have previously linked mTOR signaling to bone biology [14]; however, assessment of mTOR inhibition in bone biology in the context of osteotropic breast cancer has not been performed in detail.

In this study, low concentrations of everolimus in vitro (1, 10, and 100 nM), when used as a single treatment agent, were sufficient to potently inhibit mTOR signaling and cancer cell viability in vitro (Fig. 1a and b). Continuing with the antitumor assessment of everolimus, we tested the everolimus dose of 1 mg/kg/day in mice subcutaneously inoculated with either osteotropic B16-F10 murine melanoma cells [21] or MDA-MB-231 human breast cancer cells [22]. These tumor-bearing mice demonstrated an effective therapeutic response to treatment with everolimus, with significant decreases in the final tumor burdens in both models. First, we used the B16-F10 model to confirm potential antitumor effects of everolimus because it is an aggressive osteotropic cell line with the propensity to form osteolytic bone metastases in vivo and can be inoculated into syngeneic hosts. Putting this into context, everolimus has previously been assessed in clinical trials of patients with metastatic melanoma, often combined with inhibitors of angiogenesis [23, 24]. The therapeutic benefit of pairing everolimus in these combinations is unclear, however, with reports ranging from major responses and disease stabilization to no response at all. Obviously, there is room for improvement, and focusing on trial design and everolimus pharmacology in the future may help to elucidate the current controversies [25]. Here, we observed a potent growth-inhibitory effect of everolimus on subcutaneous B16-F10 tumors. This may be linked to possible activating mutations in mTOR [26], and these mutation-specific influences should also be considered in the context of improving the outcomes of patients with melanoma by using everolimus treatment.

With the focus returning to breast cancer, we could show that 1 mg/kg was effective at inhibiting subcutaneous tumor growth of ER-negative MDA-MB-231 cells. Of interest, this dose is tenfold lower than what is currently advised for ER-positive HER2-negative breast cancer in the clinic. However, this inconsistency could likely be explained by the administration route (intraperitoneal injection versus oral), the chosen animal model, and the cell line used. Previously, when already established subcutaneous MDA-MB-231 tumors were treated with everolimus as a monotherapy at a dose of 10 mg/kg three times weekly, the results were rather disappointing. In this case, only a modest antitumor response could be demonstrated, which was not significant [27]. Observed differences in the potency of everolimus as an antitumor agent may result from differences in the protocol, most important being the time point when treatment was commenced. Despite this, the results presented here do support other studies in which everolimus was used as a sensitizing agent to radiotherapy or chemotherapeutics against the same cell line [28–30]. Dose, route, and frequency of administration would all appear to define the potency of everolimus as an antitumor agent in breast cancer. In this context, further investigations should expand on the mechanism of how everolimus inhibits the growth and tumorigenesis of breast cancer cells. As one example, it has previously been shown that mTOR inhibition by everolimus in aromatase inhibitor-resistant breast cancer results in a concurrent induction of autophagy and a downregulation of the ER [11]. Overall, our results confirm and endorse the potent antitumor effects of everolimus in melanoma and in ER-negative breast cancer, in immunocompetent and immunocompromised models, respectively.

Both bone loss observed in hormone ablation and in the progression of breast cancer bone metastases are mediated mainly by an enhanced osteoclast activity. Previously, mTOR signaling has been shown to regulate osteoclastogenesis and osteoclast function [17, 31], and in the pathological setting, the activation of mTOR is responsible for joint destruction in arthritis [32]. In this study, 1 nM of everolimus exerted highly antiosteoclastic effects. Although osteoclasts differentiated from murine bone marrow-derived mononuclear cells appeared slightly more resistant to everolimus, they demonstrated a potent decrease in all parameters investigated at 10 and 100 nM of everolimus treatment (Fig. 2c, d). Additionally, it could be shown that everolimus inhibits the ability of mature osteoclasts to resorb bone (Additional file 3: Figure S3). Interestingly, these results are in contrast to a previous study where rapamycin (an earlier-generation mTOR inhibitor) enhanced RANKL-induced osteoclastogenesis and functionality in RAW 264.7 cells [33]. This may be explained by different proficiency and mechanisms of the mTOR inhibitors rapamycin and everolimus [34, 35]. For example, different downstream signaling effects with regard to inhibited phosphorylation S6 and AKT have been described [36]. Despite these considerations, a recent study supports our observations of everolimus as being an inhibitor of osteoclastogenesis. When investigating the effects of everolimus on the osteoclastogenesis of peripheral blood monocytes in a coculture model with the triple-negative breast cancer cell line SCP2, everolimus was effective at inhibiting osteoclastogenesis induced by SCP2-derived factors [37].

Previous studies deleting key components of mTORC1 signaling in mice have shown pro-osteoblast effects of mTORC1 signaling [38, 39], with mTOR activation promoting later stages of osteoblast differentiation in particular [40]. In contrast, studies using the mTOR inhibitors rapamycin and BEZ235 demonstrated an augmented osteoblastogenesis in human embryonic stem cells and hMSC, respectively [41, 42]. In the present study, the effects of everolimus on the differentiation of both mMSC and hMSC along the osteoblast lineage were assessed. In general, we have shown that osteoblasts were less sensitive to inhibitory effects by everolimus than osteoclasts, and some markers of osteoblastogenesis even increased when hMSC were differentiated at low doses of everolimus (Fig. 3).

When translating these in vitro osteoclast and osteoblast observations to an in vivo setting using an OVX model to mimic hormone ablation therapy and to induce bone loss, a low-dose treatment regime of everolimus (1 mg/kg/day) had potent bone-protective effects as assessed by μCT analysis. Further assessment by bone histomorphometry revealed that these positive effects were achieved primarily by an inhibition of the osteoclast-mediated bone resorption (Fig. 4b). Interestingly, whereas the numbers of osteoclasts were reduced in the bone, there was no evidence of increased anabolic activity. This is a common finding in classic antiresorptive drugs such as bisphosphonates [43]. At the effective doses, these drugs exert highly antiresorptive effects but also impair osteoblast action. However, because bone loss mediated by hormone deficiency is mediated mainly by increased osteoclastic bone resorption, the antiresorptive effects are sufficient to protect bone. In line with this assumption, we did not observe an effect of everolimus on bone when used in a nonchallenged setting. In the context of breast cancer, the mTOR signaling pathway has been shown to be involved in crosstalk with ERα signaling under estrogen stimulation [44]. With this in mind, in the absence of ER signaling, mTOR could hypothetically signal independently, promoting and enhancing the differentiation of osteoclasts.

Using the same dose that was required to suppress subcutaneous tumor growth and prevent bone loss in a hormone-deprived environment, everolimus was proven to be effective in preventing the establishment and progression of breast cancer bone metastases when mice were intracardially inoculated with the ER-negative, bone-seeking MDA-MB-231 cells (Fig. 5a–c). In line with this finding, the trabecular bone of the femur was protected against tumor-induced osteolysis and bone loss (Fig. 5e). Supporting this finding in another model of malignant bone disease prevention, treating MDA-MB-231 cells with everolimus before intratibial injection resulted in a reduction in the area of the subsequent osteolytic lesions [45]. Furthermore, at the first time point measured in the context of bone metastases arising from lung cancer, the bisphosphonate zoledronic acid was shown to be less effective than everolimus in preventing bone metastases, with 27.8% more mice developing bone lesions than in the everolimus-treated group [46]. In the intracardiac bone metastasis model, it was discovered that the number of osteoclasts in the femurs of everolimus-treated mice was significantly reduced (Additional file 5: Figure S5). This could support an additional antiosteoclastic role for mTOR in the context of cancer-induced osteolysis. However, it remains a challenge to separate the relative contributions to antitumor efficacy of everolimus as a bone-targeted agent that potentially simultaneously promotes both direct antitumor and indirect anti-bone-resorptive actions. For example, this reduction in the number of osteoclasts could be a result of an inhibition in the expression of the pro-osteoclastic factor interleukin-6 by the MDA-MB-231 cells [45]. Here, we postulate that the antibone metastatic effect of everolimus is a result of direct antitumor effects, supported by a decrease in the differentiation and activity of osteoclasts. However, further research is needed to dissect the molecular mechanisms of this intertwined effect.

## Conclusions

Overall, our results showing bone-protective effects of everolimus in the setting of a hormone-deprived environment are in agreement with the bone marker analysis of the BOLERO-2 trial [13]. Importantly, we provide further evidence that the dose required for bone protection in a hormone-deprived environment (Fig. 4) can also effectively inhibit the growth of bone metastases (Fig. 5). Not only did everolimus inhibit the growth of breast cancer bone metastases in this model but also bone destruction was significantly inhibited in the everolimus group compared with the control group. This study also provides a further rationale for considering everolimus as an antitumor agent in ER-negative breast cancer.

## Additional files

Additional file 1: Figure S1. Everolimus inhibits mTOR signaling in cancer cell lines. Quantification of Western blots shown in Fig. 1. Indicated cell lines were treated with everolimus for 24 h, and total and phosphorylated proteins were detected by Western blot analysis. The signals of phosphorylated mTOR (p-mTOR) and phosphorylated p70 S6 kinase (p-p70) were quantified and normalized to corresponding signals of GAPDH for a total of three experiments. Data were analyzed using one-way ANOVA and the Bonferroni posttest and are shown as mean ± SD (* *p* < 0.05; ** *p* < 0.01, *** *p* < 0.001). Equal volumes of DMSO used to prepare and administer everolimus treatments were used in the control conditions. (PDF 14 kb)

Additional file 2: Figure S2. Everolimus suppresses the cell viability of osteoclasts but not preosteoblasts in vitro. RAW 246.7 cells (**a**) were differentiated with RANKL for 5 days and hMSC (**b**) with an osteoblast differentiation cocktail for 8 days before the addition of everolimus for 2 days. The CellTiter-Blue® assay was then performed to assess viability. Data were analyzed using one-way ANOVA and the Bonferroni posttest and are shown as mean ± SD (*** *p* < 0.001). Equal volumes of DMSO used to prepare and administer everolimus treatments were used in the control conditions. (PDF 1346 kb)

Additional file 3: Figure S3. Everolimus inhibits the bone-resorbing activity of osteoclasts. Murine bone marrow-derived mononuclear cells were differentiated to osteoclasts on bone slices in vitro before being treated with everolimus at concentrations of 1, 10, and 100 nM for 5 days in total. On day 5, supernatants were collected and analyzed for the levels of the bone resorption marker collagen type I cross-linked C-telopeptide (CTx). Data were analyzed using one-way ANOVA and the Bonferroni posttest, and significance between the control and everolimus concentrations is denoted (** *p* < 0.01). (PDF 9 kb)

Additional file 4: Figure S4. Standard histological sections of TRAP staining in the femur. Representative images of an OVX control-treated femur and an everolimus-treated femur stained for TRAP (**a**, ×2.5 magnification, scale bar 200 μm; **b**, ×20 original magnification, scale bar 50 μm). (PDF 3572 kb)

Additional file 5: Figure S5. Everolimus reduces the number of osteoclasts in the femurs of mice bearing bone metastases. Quantification and representative TRAP staining of osteoclasts (*red*) in the femurs of mice from the intracardiac bone metastasis model (Fig. 5). Original magnification × 20, scale bar 50 μm. Data are shown as mean ± SD and were analyzed using Student’s *t* test (* *p* < 0.05). *B* Bone, *T* Tumor. (PDF 2263 kb)

## Abbreviations

ACTB/Actb: β-Actin
ALP/Alp: Alkaline phosphatase
ANOVA: Analysis of variance
ATCC: American Type Culture Collection
BFR/BS: Bone formation rate per unit of bone surface
BMD: Bone mineral density
BMP: Bone morphogenetic protein
BV/TV: Bone volume over total volume
Ctsk: Cathepsin K
CTx: Collagen type I cross-linked C-telopeptide
DMSO: Dimethyl sulfoxide
ER: Estrogen receptor α
EV: Everolimus
FCS: Fetal calf serum supreme
GAPDH: Glyceraldehyde 3-phosphate dehydrogenase
HA: Hydroxyapatite
hMSC: Human mesenchymal stem cells
hu: Human
IgG: Immunoglobulin G
M-CSF: Macrophage colony-stimulating factor
μCT: Micro-computed tomography
mMSC: Murine mesenchymal stem cells
mRNA: Messenger RNA
mTOR: Mammalian target of rapamycin
mu: Murine
OCN/Ocn: Osteocalcin
OPG/Opg: Osteoprotegerin
OVX: Ovariectomy/ovariectomized
P/S: Penicillin/streptomycin
PFA: Paraformaldehyde
qRT-PCR: Quantitative real-time polymerase chain reaction
RANKL: Receptor activator of nuclear factor κB ligand
RUNX2/Runx2: Runt-related transcription factor 2
SDS: Sodium dodecyl sulfate
Tb.N: Trabecular number
Tb.Sp: Trabecular separation
TRAP: Tartrate-resistant acid phosphatase
