# Supplementary figures and images for: Concurrent antitumor and bone-protective effects of everolimus in osteotropic breast cancer
Source: Breast Cancer Res. 2017 Aug 9;19:92. doi: 10.1186/s13058-017-0885-7 (PMC5551016; doi:10.1186/s13058-017-0885-7)

B16-F10

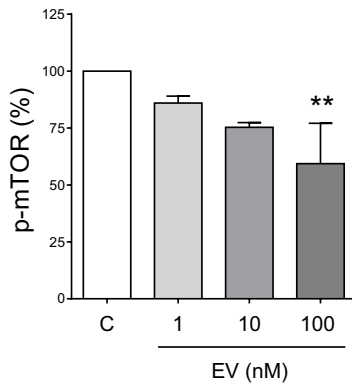

MDA-MB-231

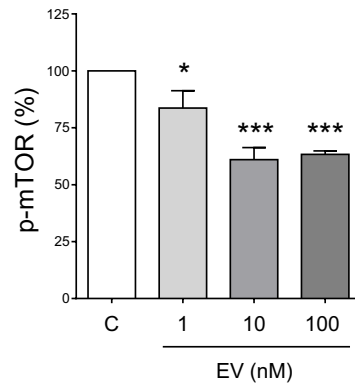

MCF-7

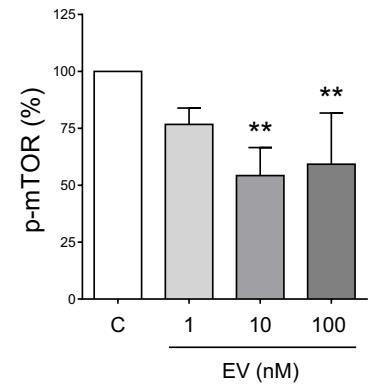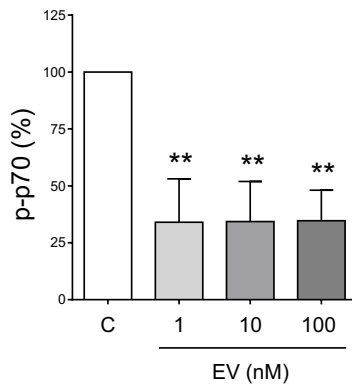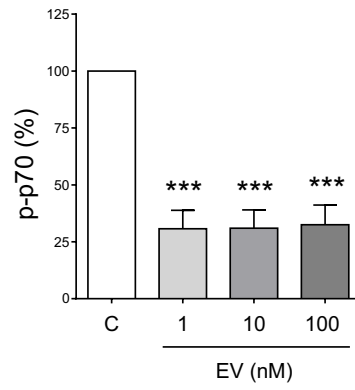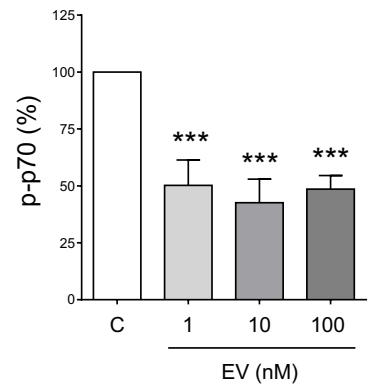

Supplement: Supplementary file 1 — Everolimus inhibits mTOR signaling in cancer cell lines. Quantification of Western blots shown in Fig. 1. Indicated cell lines were treated with everolimus for 24 h, and total and phosphorylated proteins were detected by Western blot analysis. The signals of phosphorylated mTOR (p-mTOR) and phosphorylated p70 S6 kinase (p-p70) were quantified and normalized to corresponding signals of GAPDH for a total of three experiments. Data were analyzed using one-way ANOVA and the Bonferroni posttest and are shown as mean ± SD (* p < 0.05; ** p < 0.01, *** p < 0.001). Equal volumes of DMSO used to prepare and administer everolimus treatments were used in the control conditions. (PDF 14 kb) [file 13058_2017_885_MOESM1_ESM.pdf]

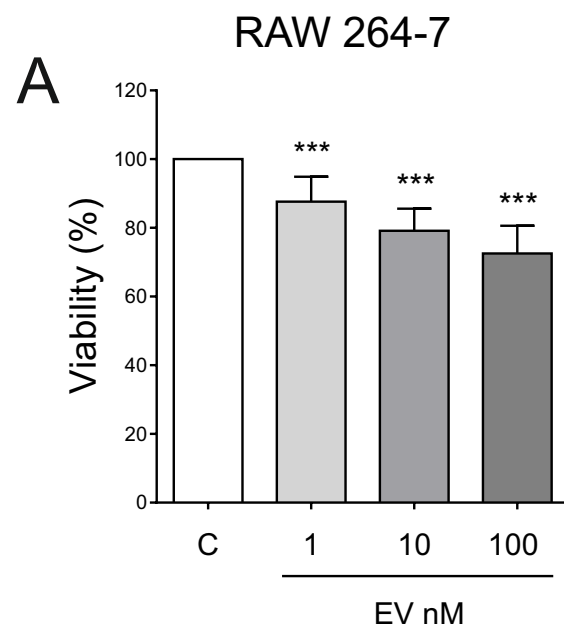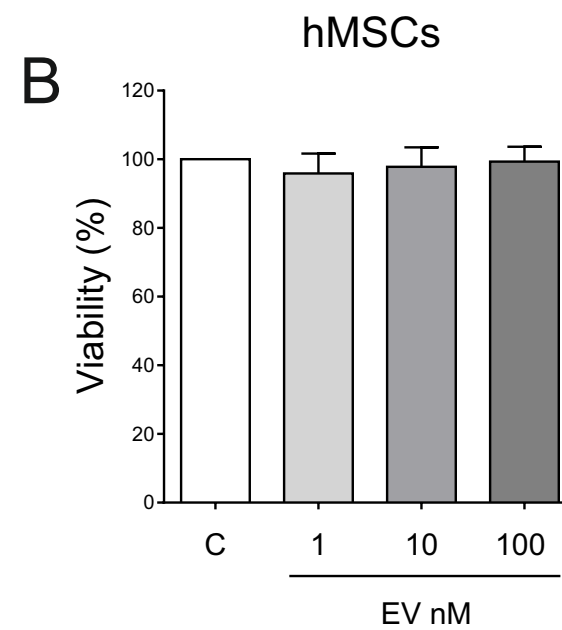

Supplement: Supplementary file 2 — Everolimus suppresses the cell viability of osteoclasts but not preosteoblasts in vitro. RAW 246.7 cells (a) were differentiated with RANKL for 5 days and hMSC (b) with an osteoblast differentiation cocktail for 8 days before the addition of everolimus for 2 days. The CellTiter-Blue® assay was then performed to assess viability. Data were analyzed using one-way ANOVA and the Bonferroni posttest and are shown as mean ± SD (*** p < 0.001). Equal volumes of DMSO used to prepare and administer everolimus treatments were used in the control conditions. (PDF 1346 kb) [file 13058_2017_885_MOESM2_ESM.pdf]

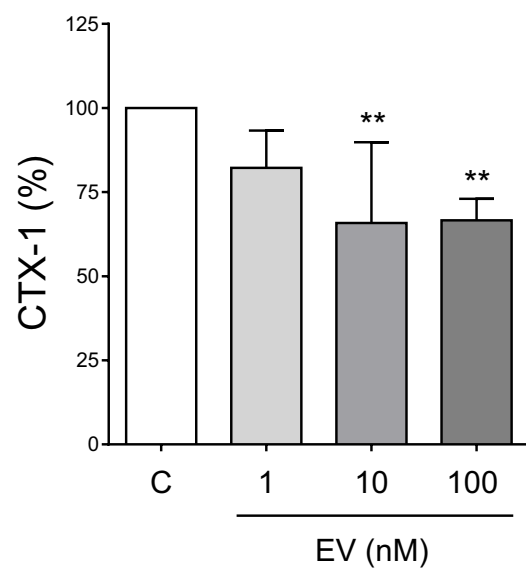

Supplement: Supplementary file 3 — Everolimus inhibits the bone-resorbing activity of osteoclasts. Murine bone marrow-derived mononuclear cells were differentiated to osteoclasts on bone slices in vitro before being treated with everolimus at concentrations of 1, 10, and 100 nM for 5 days in total. On day 5, supernatants were collected and analyzed for the levels of the bone resorption marker collagen type I cross-linked C-telopeptide (CTx). Data were analyzed using one-way ANOVA and the Bonferroni posttest, and significance between the control and everolimus concentrations is denoted (** p < 0.01). (PDF 9 kb) [file 13058_2017_885_MOESM3_ESM.pdf]

A

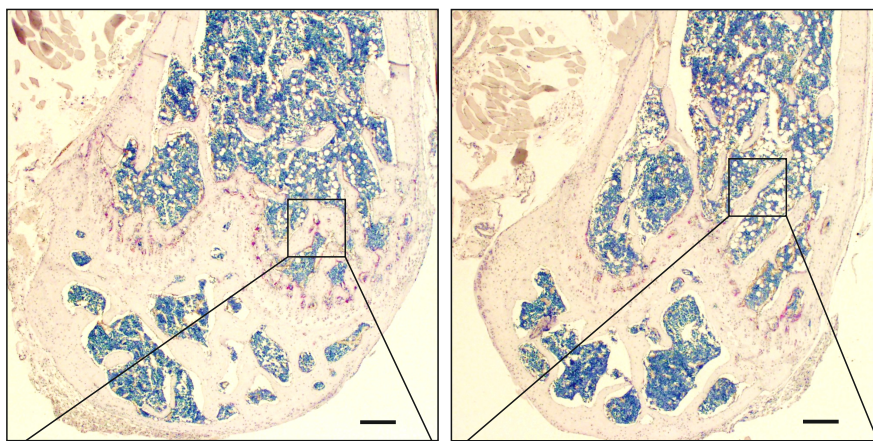

B

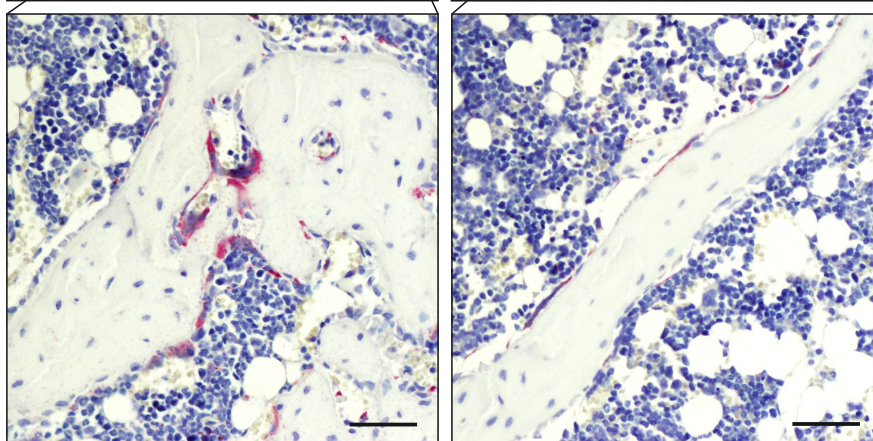

Control OVX

EV OVX

Supplement: Supplementary file 4 — Standard histological sections of TRAP staining in the femur. Representative images of an OVX control-treated femur and an everolimus-treated femur stained for TRAP (a, ×2.5 magnification, scale bar 200 μm; b, ×20 original magnification, scale bar 50 μm). (PDF 3572 kb) [file 13058_2017_885_MOESM4_ESM.pdf]

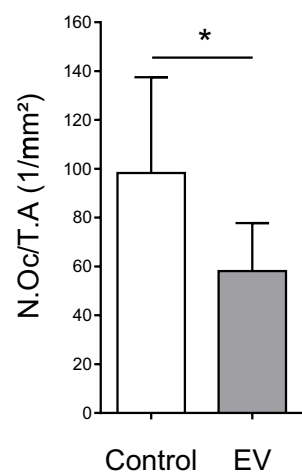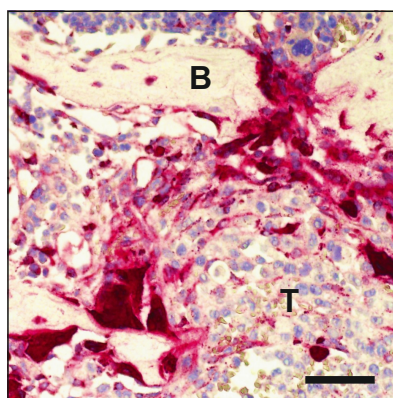

Control

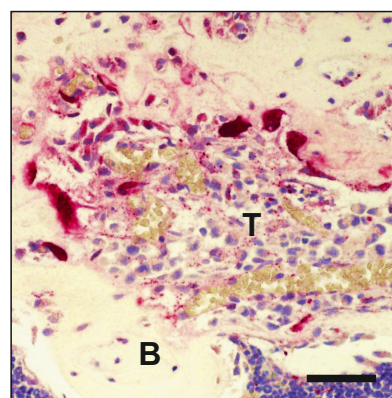

EV

Supplement: Supplementary file 5 — Everolimus reduces the number of osteoclasts in the femurs of mice bearing bone metastases. Quantification and representative TRAP staining of osteoclasts (red) in the femurs of mice from the intracardiac bone metastasis model (Fig. 5). Original magnification × 20, scale bar 50 μm. Data are shown as mean ± SD and were analyzed using Student’s t test (* p < 0.05). B Bone, T Tumor. (PDF 2263 kb) [file 13058_2017_885_MOESM5_ESM.pdf]
